# Supplementary material for: Resolving forebrain developmental organisation by analysis of differential growth patterns
Source: Nat Commun. 2025 Dec 21;17:901. doi: 10.1038/s41467-025-67623-6 (PMC12830808; doi:10.1038/s41467-025-67623-6)
Supplement: Supplementary file 1 — Supplementary information [file 41467_2025_67623_MOESM1_ESM.pdf]

Supplementary Materials:
Resolving forebrain developmental organisation by analysis of differential growth patterns

Elizabeth Manning[1-3], Kavitha Chinnaiya[1-3], Caitlyn Furley[1-3], Dong Won Kim[4,5], Seth Blackshaw[6-11], Marysia Placzek[1-3]* and Elsie Place[1-3]*

Corresponding authors: m.placzek@sheffield.ac.uk, e.place@sheffield.ac.uk.

Containing:
Figs. S1 to S19
Supplementary references 1 - 11

Movies S1 to S4 are provided separately

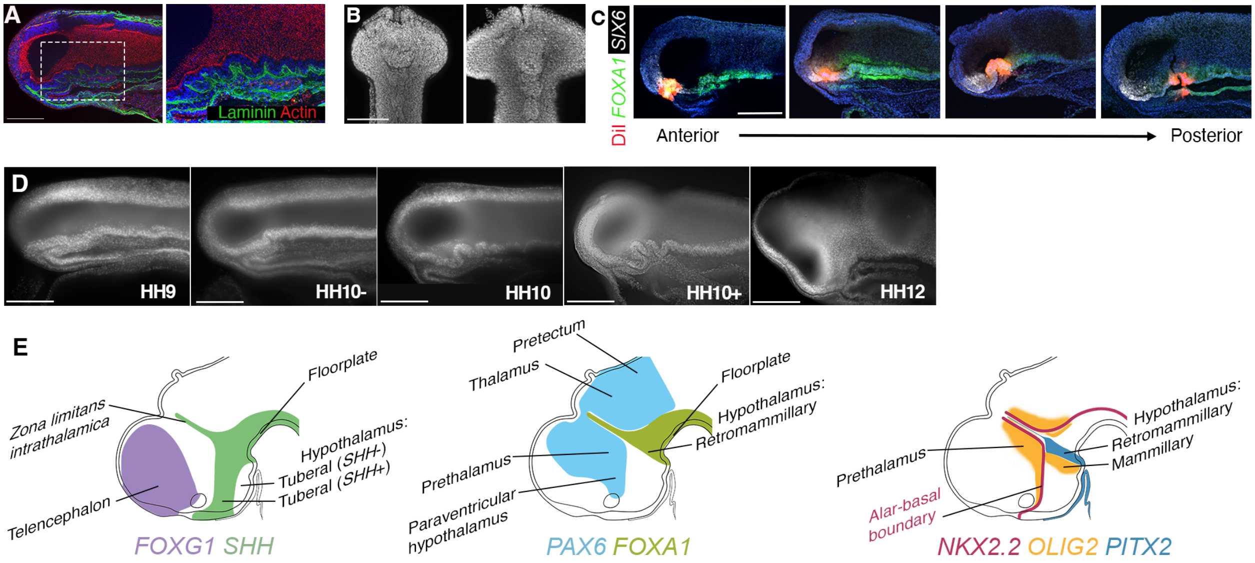

fig. S1

Morphological and genetic landmarks at HH10 and HH20

(A) Hemisected internal view of HH10 embryo immunolabelled for laminin and stained with phalloidin (to reveal Actin) reveals midline epithelial folds underlain by basement membrane. Higher resolution of boxed area shown to right. **(B)** HH10.0 (left hand panel) and HH10.2 (right hand panel) isolated neuroepithelia, DAPI-stained, ventral views. Midline epithelial folds increase in number and A-P extent from HH10.1 to HH10.2 but the anterior-most fold remains the most prominent. **(C)** Hemisected internal views of HH10 embryos analysed by HCR for expression of *FOXA1* (green) and *SIX6* (white) after DiI targeted to consecutive ventral midline regions of zones 1 or 2 confirms accuracy of injections relative to the anterior fold. **(D)** Hemisected internal views of HH9-HH12 chicks, DAPI-labelled. Epithelial folds form, then resolve between these developmental stages. **(E)** Selected regional marker gene expression patterns. Scale bars - 250 µm

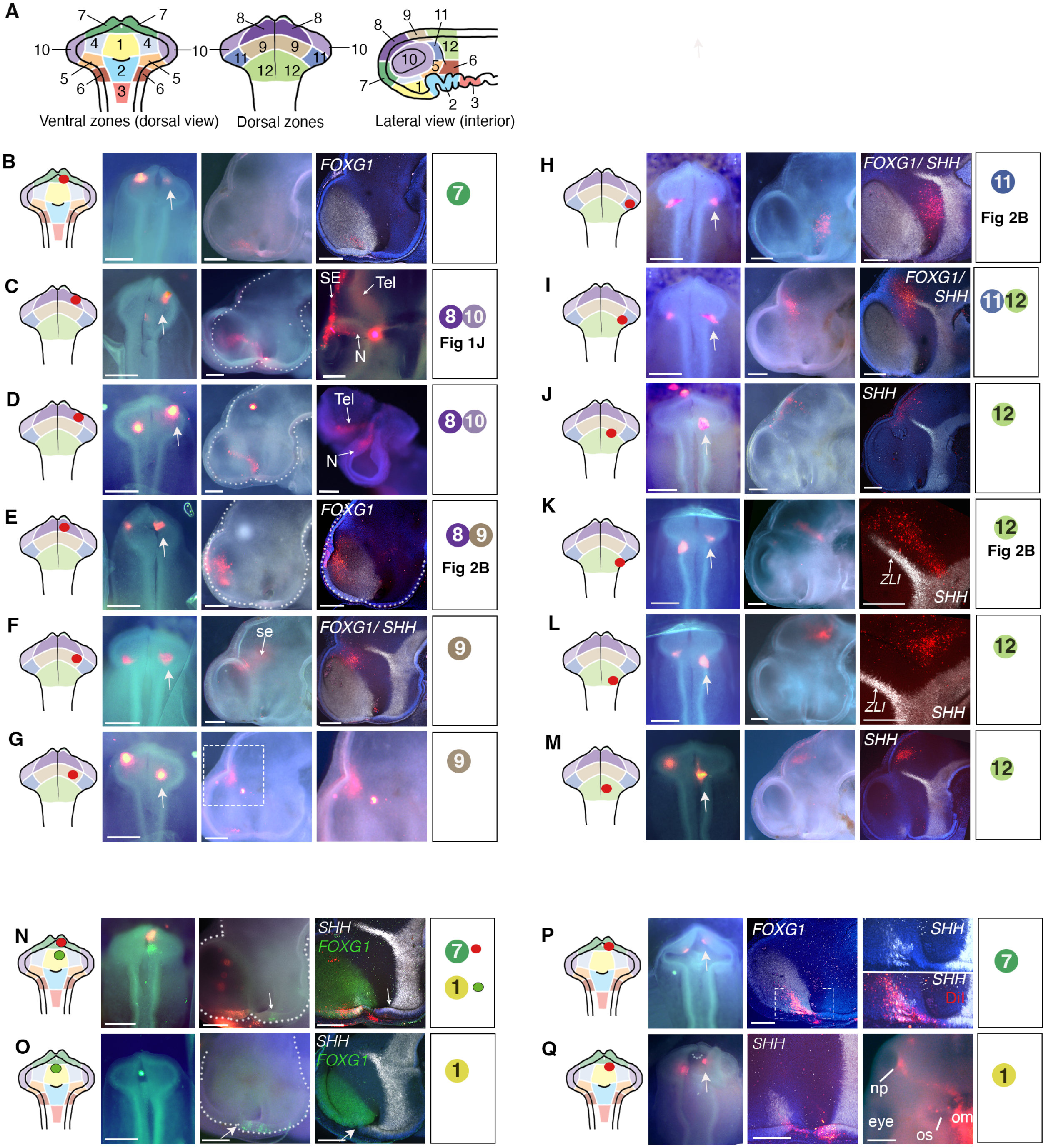

fig. S2

Regionally distinct patterns of anisotropic growth in the dorsal anterior neural tube

A) HH10 forebrain, depicting targeted zones . **(B-O)** From left to right: panels 1 - schematic injection site(s); panels 2 - *in ovo* images of initial injection. White arrows point to injections shown at HH20 (panels 3, hemisected internal views). Panels 4 - hemisected internal views after HCR in situ to detect genes indicated (**B**, **E-F**, **H-O**), or alternative views to aid with visualising particular growth lines (**C-D** - ventral views; **G** - increased magnification). Panels 5 - regions

mapped and if shown in main figures. All are presented as right hand sides to assist comparison. **(B)** Cells labelled lateral to the anterior neuropore in zone 7 label the anterior telencephalon. (**C-D)** DiI-labelled growth lines from zone 8/10 injection stretch from telencephalon to optic stalk (panels 3) and into nasal eye (panels 4; ventral views); **(E)** Dorsal DiI labelling of zone 12 results in a more isometric expansion. **(F-G)** Zone 9 injections give rise to growth line skirting telencephalon and prethalamic eminence; arrow in **(F)** points to DiI in underlying tissue (**G**) Same embryo as (**D**). (**H**) Isometric expansion of region 11 to form prethalamus/ PV hypothalamus. (**I-M**) Injections targeted to different points in zone 12 injections label different parts of the thalamus. (**K**) DiI immediately posterior to ZLI results in highly D-V elongated growth lines. (**L**) Same embryo as K. (**N-O**) DiI (red) and DiO (green) **(N)** or just DiO labelling **(O)** around ventral limit of anterior neuropore. In **(N)** DiI lateral to neuropore (zone 7) labels telencephalic *FOXG1*$^{+ve}$ midline; in (**N, P**) DiO ventral/posterior to neuropore (area 1) labels anterior hypothalamic *SHH*$^{+ive}$ midline (Hm1, Fig. 1H) Arrows point to anterior limit of DiO expression (does not show after HCR processing). (**P-Q**) embryos with dorsal tissue removed to better visualise ventral cells. Lateral area 7 (**P**) injections label *FOXG1/SHH*$^{+ve}$ subpallium. Lateral area 1/7 boundary (**Q**) labels *SHH*$^{+ve}$ anterior hypothalamus and crosses the optic midline to *SHH*$^{+ve}$ subpallium. (**Q**) panel 4 shows ventral view at HH20 with DiI connecting nasal placode to the optic midline DiI (not all DiI is in the neuroectoderm). N - Nasal retina, NP - nasal placode, OS - optic stalk, OM - optic midline, SE - surface ectoderm, T - Temporal retina, Tel - Telencephalon. Scale bars - 250 µm.

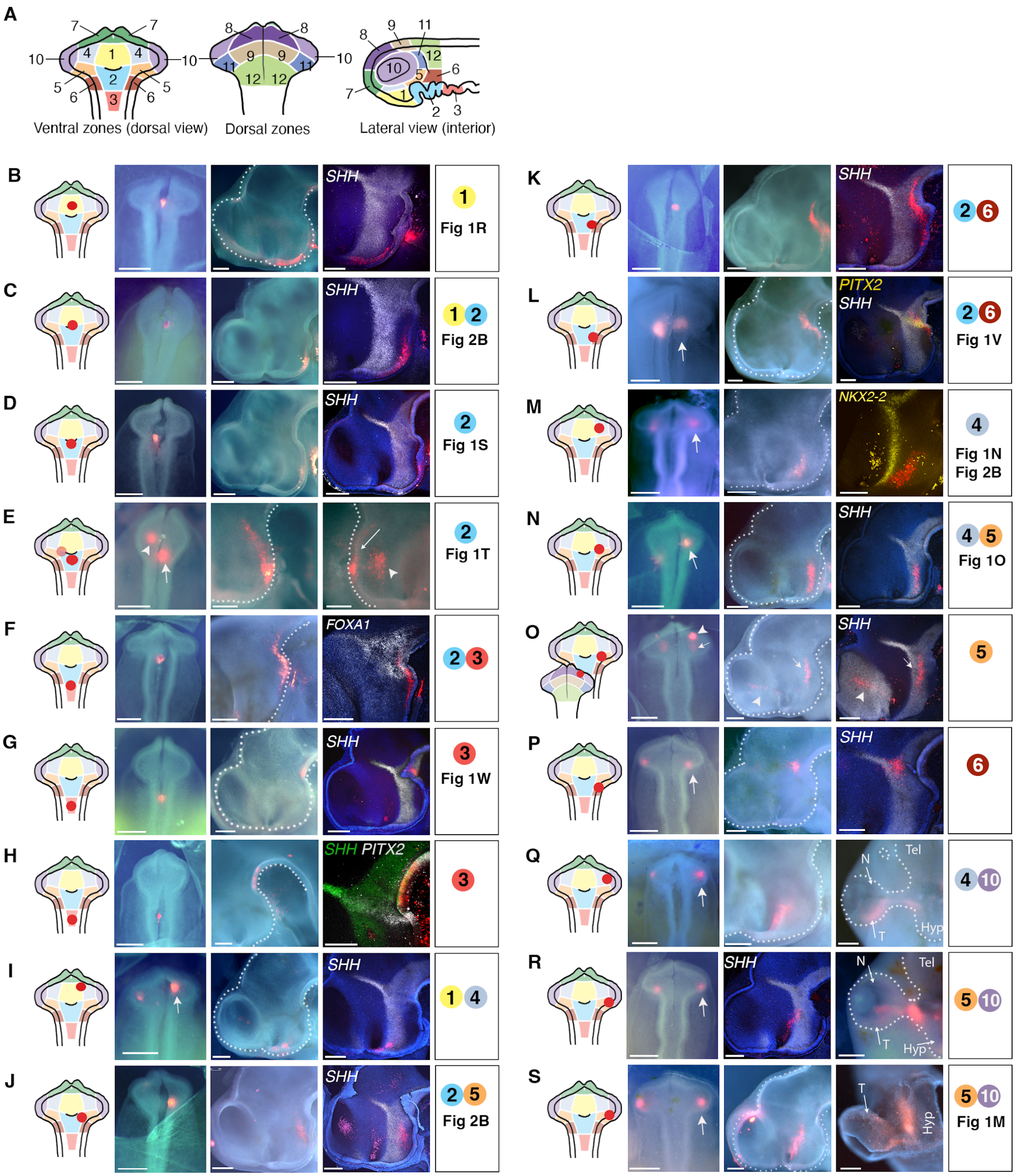

fig. S3

Regionally distinct patterns of anisotropic growth in the ventral anterior neural tube

A) HH10 forebrain, depicting the 12 zones targeted for injection. From left to right: panels 1 show schematic injection site(s); panels 2 show *in ovo* images of initial injection(s). White arrows point to injections shown at HH20 (panels 3, hemisected internal views), arrowheads

indicate secondary injection panel 1, injection site schematic. Panel 2, *in ovo* images of initial injection, white arrows indicate injection displayed in panel 3, arrowheads indicate secondary injection. Panel 2 in (**R**) shows sample after HCR for *SHH*. Panel 3 shows hemisected internal views (**B-Q, S**) or HCR processed sample (**R**). Panel 4, hemisected internal views after HCR in situ to detect genes indicated (**B-D, F**-**P**), the other side of the embryo (**E**) or outer views showing eyes (**Q, R -** ventral view, **S** - posterior view), flipped horizontally, to aid comparison with hemiviews. Panel 5 indicates injection area and whether sample has been used in main figures. (**B-H**) Injections along the midline at anterior to posterior positions, zones 1-3. Growth lines retain their A-P position along the midline, with wider, slightly lateral injections forming a V shape (**E, F**). (**I-L**) Border injections of medial and lateral zones (1,2,3 /4,5,6) produce grow lines that stretch along the A-P axis, posteriorly, curving round the *PITX2*$^{+ve}$ region (**K, L**). Medial zone 4 and 5 injections stretch along the A-P axis in the *SHH*$^{+ve}$ hypothalamus (**M-O**), whilst zone 6 injections label a small tricorn shape at the base of the ZLI (**P**). Lateral zone 4 and 5 injections cross the *NKX2.2*$^{+ve}$ ABB into the *SHH*$^{-ve}$ PV hypothalamus, stretching towards the optic stalk and through to the temporal eye (**Q-S**). More posterior injections (zone 5) cross at a more posterior point and label the more medial optic stalk and temporal eye, whilst zone 4 injections cross the ABB closer to the eye and label more lateral temporal eye (compare **Q** and **R,** panel 4 shows ventral views of HH20 embryos). Hyp - hypothalamus, N - Nasal retina, NP - nasal placode, T - Temporal retina, Tel - Telencephalon. Scale bars - 250 µm.

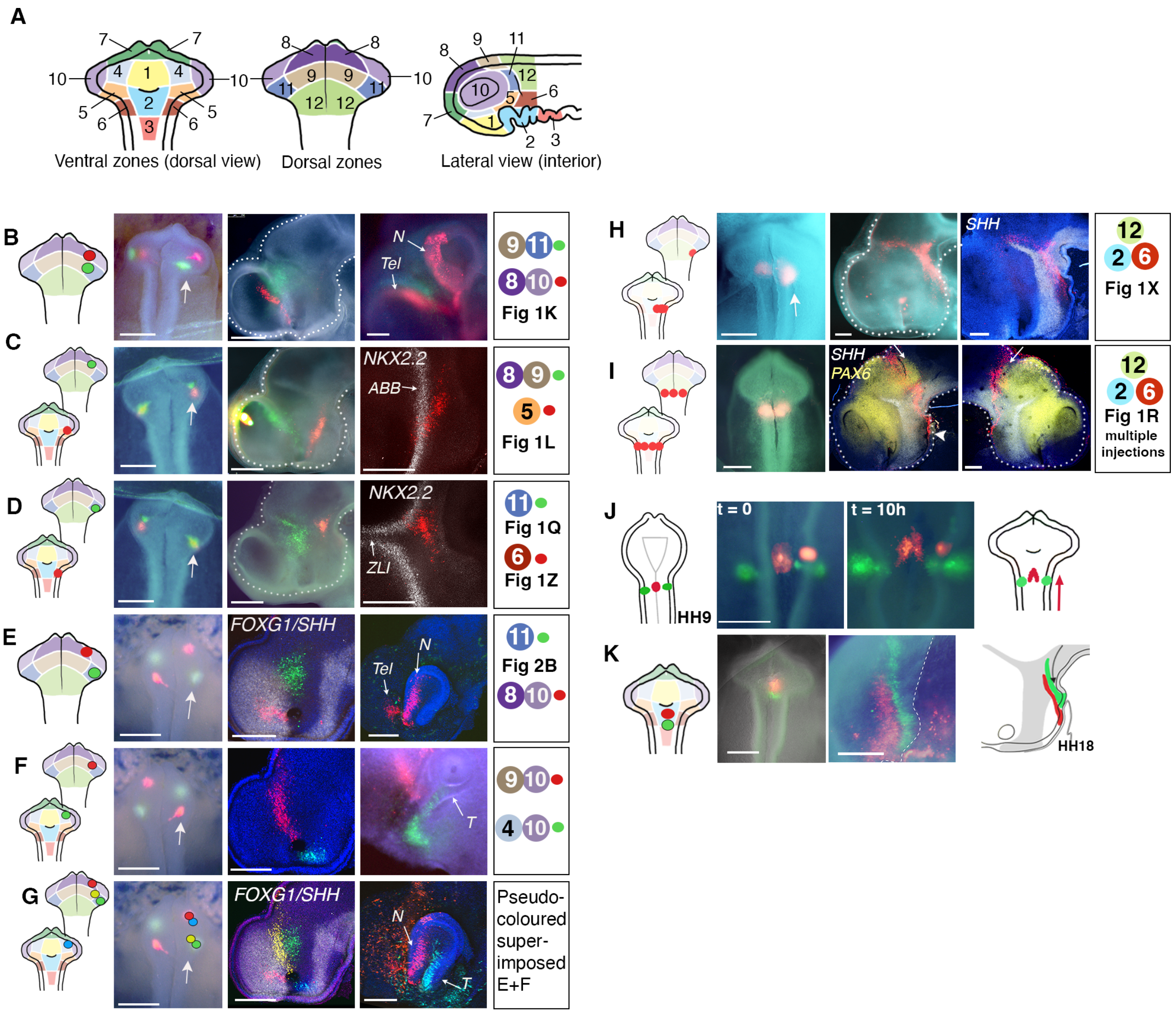

fig. S4

Comparing regionally distinct patterns of anisotropic growth via multiple injection sites. Differences in growth patterns can be highlighted by labeling multiple sites in one embryo. **A)** Ventral, dorsal and lateral views of the HH10 forebrain, depicting the 12 zones targeted for injection. From left to right (**B-I**), panel 1, injection site schematic. Panel 2, *in ovo* images of initial injection, white arrows indicate injection displayed in panels 3/4. Panel 3 shows hemisected internal views either before HCR processing or after (**E, G, I**). Panel 4 shows outer eye view, flipped horizontally to aid interpretation (**B, E-G**), hemisected internal views after HCR in situ to detect genes indicated (**C-D, H-I**), or the left side of the embryo (**I**). DiI (red) and DiO (green) injections are viewed after 48 hours (**B-D, H, I**) or 24 hours (**E-G**). **(B)** DiI-labelled growth line stretches from telencephalon to eye (panel 3) and into nasal eye (panel 4; anterior view), DiO shows uniform expansion of prethalamic territory. (**C**) DiO growth line stretches from the dorsal telencephalon to the dorsal posterior (nasal) optic stalk, whilst DiI line stretches from the posterior hypothalamus to the dorsal ventral (temporal) optic stalk. Together the DiI

and DiO growth lines create a triangle that outlines the prethalamus and PV hypothalamus. **(D)** Same embryo as in (**C**). Zone 11 (DiO) labels the prethalamus (compare to V shape in (**C**) above), and zone 6 (DiI) injection forms a tricorn shape close to the ABB (marked by *NKX2-2*) and the ZLI (flanked by *NKX2-2*). (**E)** Zone11 (DiO) labels prethalamus/PV hypothalamus, zone8/10 (DiI) labels *FOXG1*$^{+ve}$ telencephalon and nasal eye, as shown in panel 4 (outer view of eye). **(F)** Same embryo as in (**E**). Zone 8/9 boundary (DiI) stretches along the border of the *FOXG1*$^{+ve}$ telencephalon (compare with HCR in (**E**). Zone 4 injection stretches from anterior hypothalamus along the optic stalk, as shown in ventral view in panel 4. **(G) (E+F)** superimposed, with pseudocolour to (**F**) injections. Zone 8 DiI (Red) and zone 4 DiO (blue) link the telencephalon to the nasal eye and the hypothalamus to the temporal eye respectively (panel 4). **(H)** large injection over multiple zones. Dorsal label runs parallel to the ZLI in the thalamus with cells labeled at the dorsal tip of the ZLI spilling over into the prethalamus; ventral label extends at an angle towards the tuberal hypothalamic midline. The direction of predominant growth changes at the ABB. (**I**) Multiple injections at different D-V positions at a narrow transverse level. Left and right sides of the HH20 embryo shown, left side not horizontally flipped. Dorsal label runs along the D-V axis of the *PAX6*$^{+ve}$ pretectum (right side) or pretectum-midbrain boundary (left side) (arrows). Ventral label extends more anteriorly and enters the tuberal *SHH*$^{-ve}$ hypothalamus (arrowhead). **(J)** Schematic shows dorsal view of HH9+ embryo after targeting DiI to the ventral midline and DiO to more lateral regions at the same transverse level. *In ovo* images of injected region (anterior to top), shown at t = 0 and following 10 hours incubation (to ~HH11-12). Midline DiI-labelled cells move anterior relative to lateral DiO-labelled cells, forming a V shape, depicted in schematic, panel 4. **(K)** Wide injection of DiO immediately posterior to injection of DiI at the ventral midlines of zones 2 and 3 Resulting growth lines at HH18 (panel 3) form a nested V-shape shown as schematic in panel 4. N - Nasal retina, T - Temporal retina. Scale bars - 250 µm.

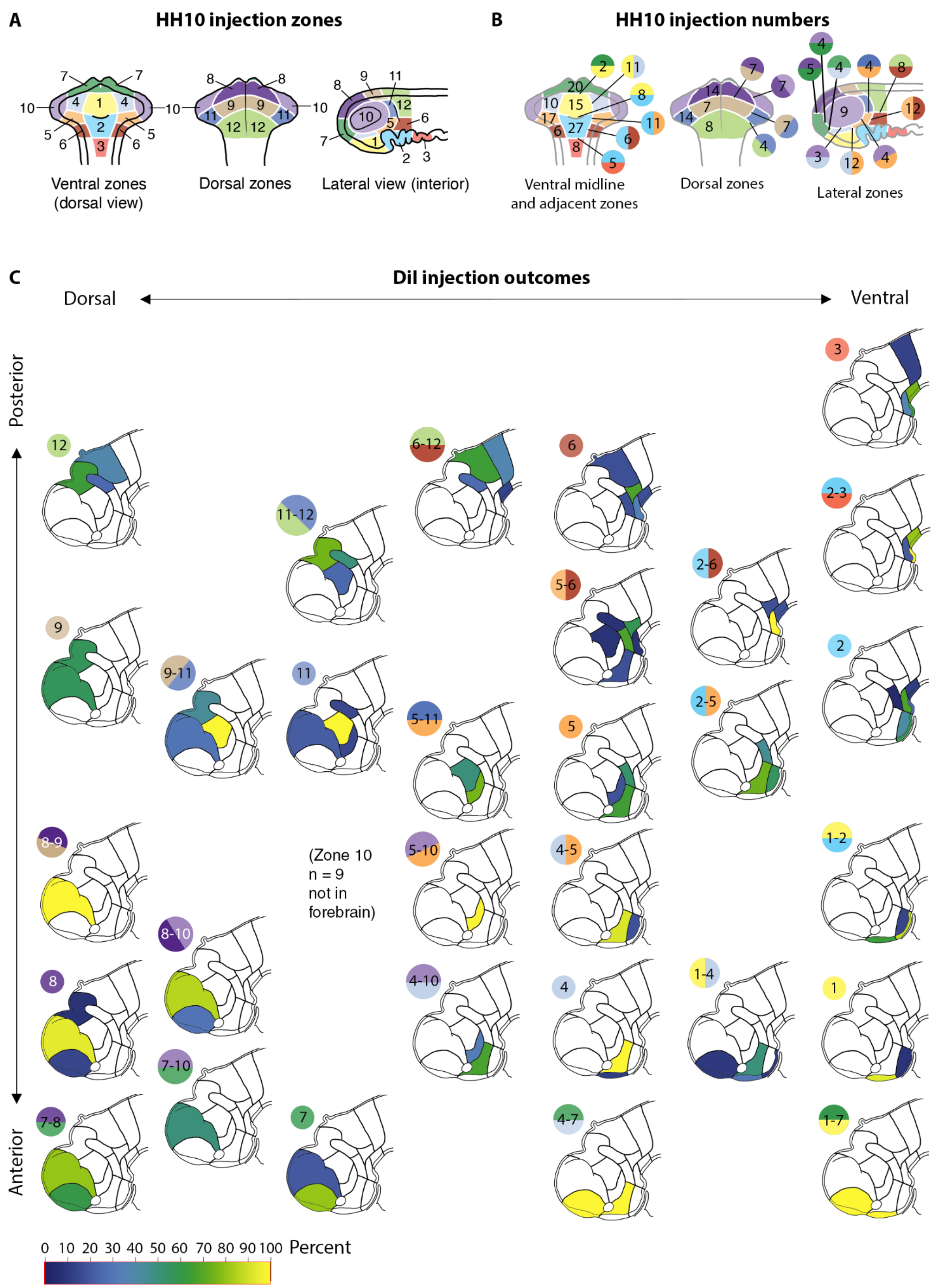

fig. S5

Quantification of fate mapping results

A) HH10 forebrain divided into 12 zones targeted for injection. (**B**) The same regions showing the number of injections recorded at each site. Injections that fell within a single region are

numbered on the embryo, injections overlapping two regions are shown in colour-coded circles. (**C**) Results of DiI/DiO injections at the twelve areas and their intersections. For each injection position (indicated by circles), the seventeen HH20 outcome regions (as per Fig. 1H) are coloured according to the heatmap (bottom left), indicating the proportion of injections that resulted in labelling of that region; white = no examples recorded. Total number of injections included in quantitative analysis = 290. Source data are provided as a Source Data file.

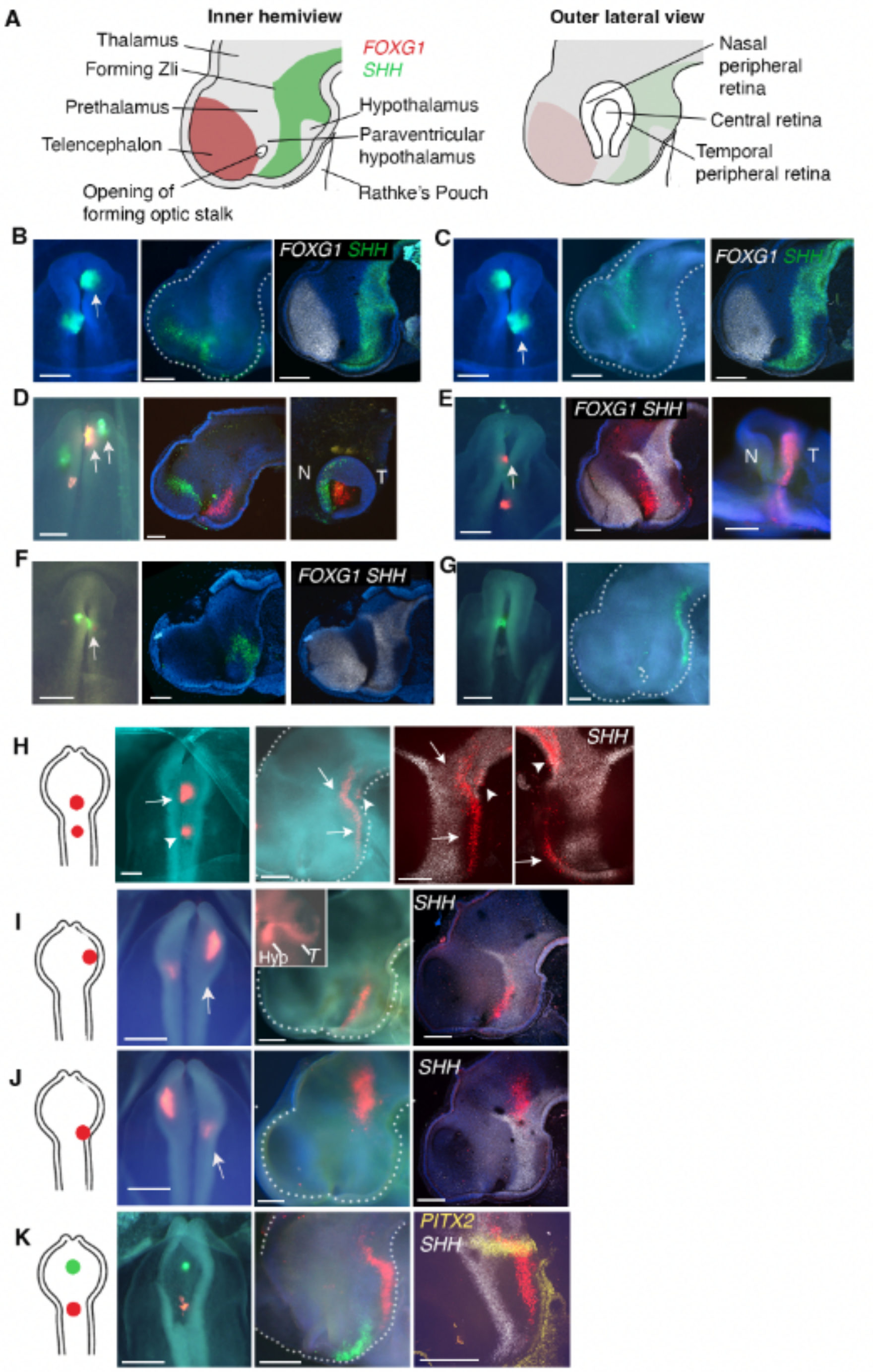

fig. S6

Growth lines resulting from HH7-9 injections

(A) Forebrain regions and marker genes at HH15. Inner hemiview (left) and outer, eye view (right). (**B-G)** Embryos injected at HH7-8 and developed for 48 hours to HH15-16. Initial injection shown *in ovo* to left. HH15-16 embryos are shown before and after HCR processing. Note, DiO does not survive HCR processing. (**B)** Anterior dorsal injection (arrow) forms elongated growth line in telencephalon. *FOXG1* (white) and *SHH* (green) shown on the same

sample. **(C)** Left side of same embryo shown in (B), displayed as right side. The more posterior DiO injection (arrow) labels a large area encompassing both prethalamus and PV hypothalamus. **(D)** Double-labelled embryo shows growth lines from ventral anterior and dorsal anterior regions (arrows) stretching into the eye. Views of isolated neuroepithelium (middle, right panels) show telencephalic growth line connecting to the nasal retina, and hypothalamic growth line connecting to the central retina. **(E)** Growth line from a ventro-lateral injection stretching between the PV hypothalamus and temporal eye. **(F)** A large medio-ventral injection at HH8 labels the tuberal hypothalamus. Most of the growth line slopes anteroventrally, with a smaller dorsal part directed towards the eye. **(G)** A midline injection at HH8 becomes highly elongated along the A-P axis, from the tuberal hypothalamic midline to regions just lateral to the diencephalic midline. (**H-K**) HH9 injected embryos developed to HH18. Schematic to left shows position of injection, next to *in ovo* images at HH9. Panels 3 and 4 show internal hemiviews at HH18, before (panel 3) or after (panel 4) HCR labelling to detect *SHH* (H-J) or *SHH* and *PITX2* (K). Panel 5 in (H) shows left side of same embryo. (**H**) Injections in the midline become highly elongated along the A-P axis, displaying similar V-shape patterns to those detected at HH10. Posterior injection marked by arrowhead, labels diencephalic floor plate. (**I**) A ventrolateral injection at HH9 extends from the posterior hypothalamus to the temporal eye (insert shows ventral view of eye). (**J**) Same embryo as (I). A lateral injection labels the ventral thalamus. (**K**) Double injection (DiI - red, DiO - green, dorsal tissue removed at HH9 to aid visualisation). The anterior ventral DiO injection labels the anterior hypothalamus; the posterior DiI labels the tuberal hypothalamus, crossing over at the *PITX2*$^{+ve}$ retromammillary hypothalamus. Both growth lines are in the midline anteriorly but form a V shape, extending to more lateral regions at their posterior end, similar to the growth patterns detected at HH10. Hyp - Hypothalamus, N - Nasal retina, T - Temporal retina. Scale bars - 250 µm.

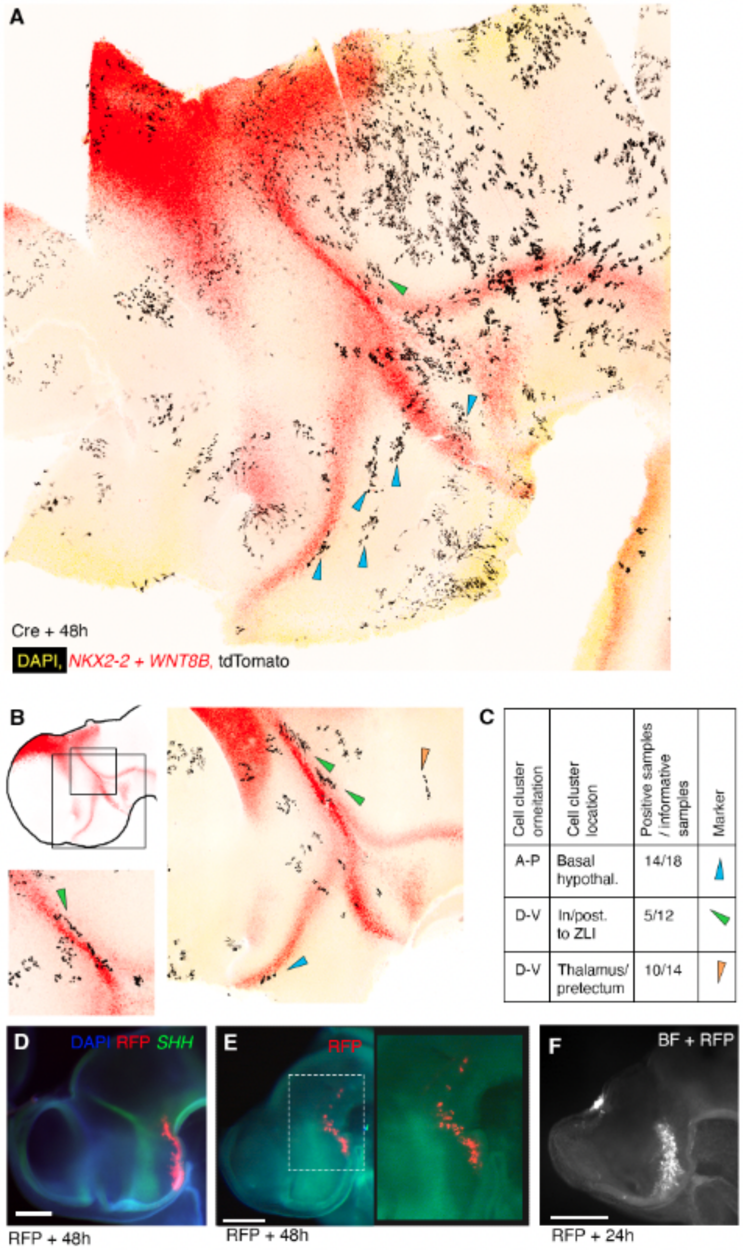

fig. S7

Cre-recombination lineage tracing and RFP electroporation confirms anisotropic growth patterns

(A, B) Flat mounted neurectoderm from Chameleon chick brains (*1*) following injection of TAT-Cre recombinase into the lumen of the head at HH9. Following 48 hours of development (to ~HH18), samples were processed for *NKX2.2* and *WNT8B* expression by HCR. Resulting tdTomato^{+ve} clones from Cytbow cassette recombination are shown in black. Blue arrowheads indicate clones that have extended along the A-P axis in the basal hypothalamus, green arrowheads indicates D-V expansion posterior to the ZLI, and orange arrowhead indicates D-V

expansion in the thalamus/ pretectum. Boxes in schematic in (B) indicate areas shown below and to the right. **(C)** Quantification of the three patterns shown. Positive samples - the number of specimens containing cell group(s) showing this pattern. Informative samples - the number of specimens containing well-spaced cell groups in the specified region, that could therefore be assessed for their shape/orientation. **(D-F)** Targeted electroporation of an RFP construct at HH10 leads to distributions of labelled cells within the basal hypothalamus that resemble the shapes of growth lines seen with DiI/DiO labelling. Scale bars - 250 μm.

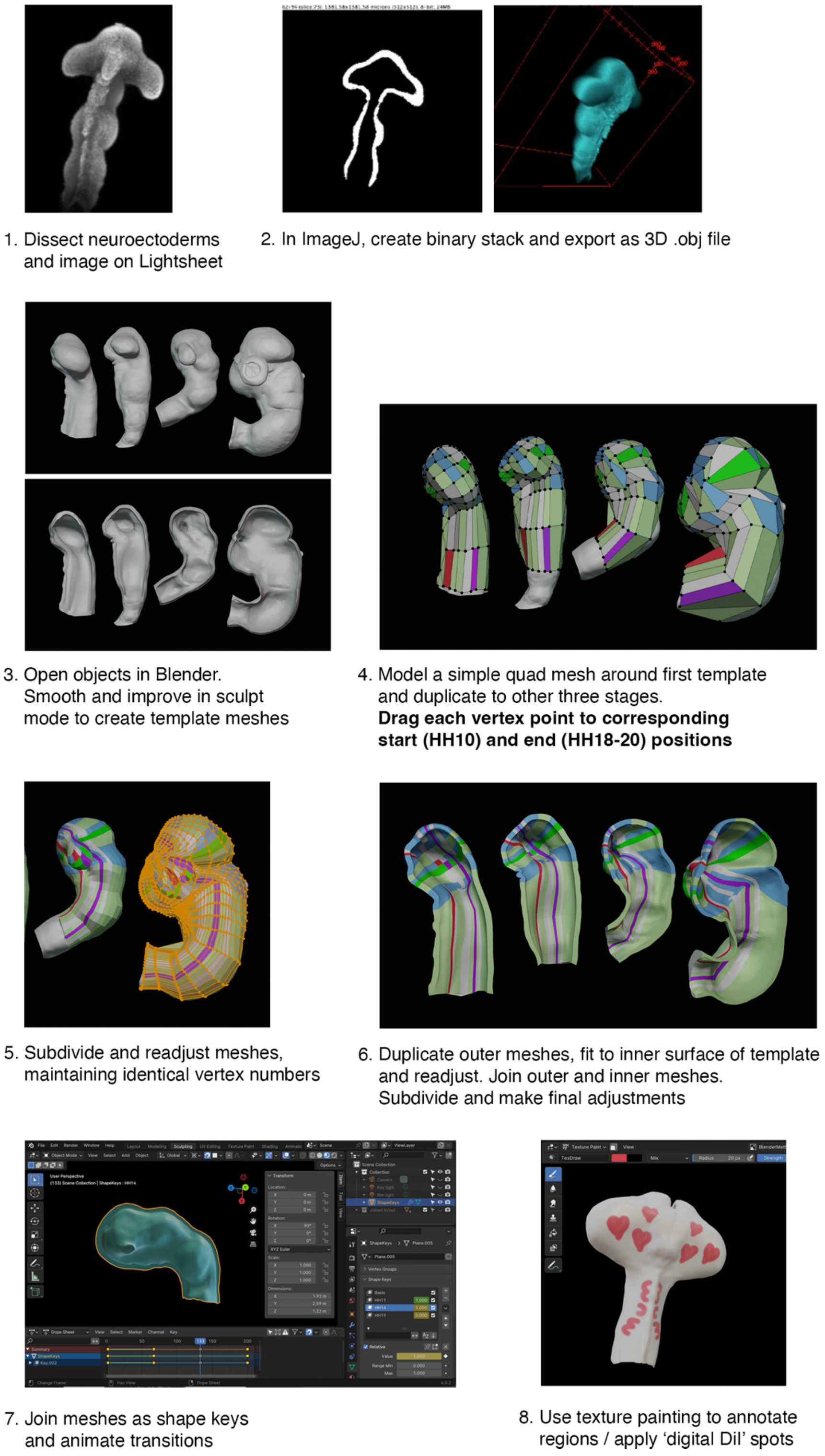

fig. S8

Building a 4D growth model

Stages involved in building the 4D forebrain model using Blender software (see methods for details). Scale bars - 250 µm.

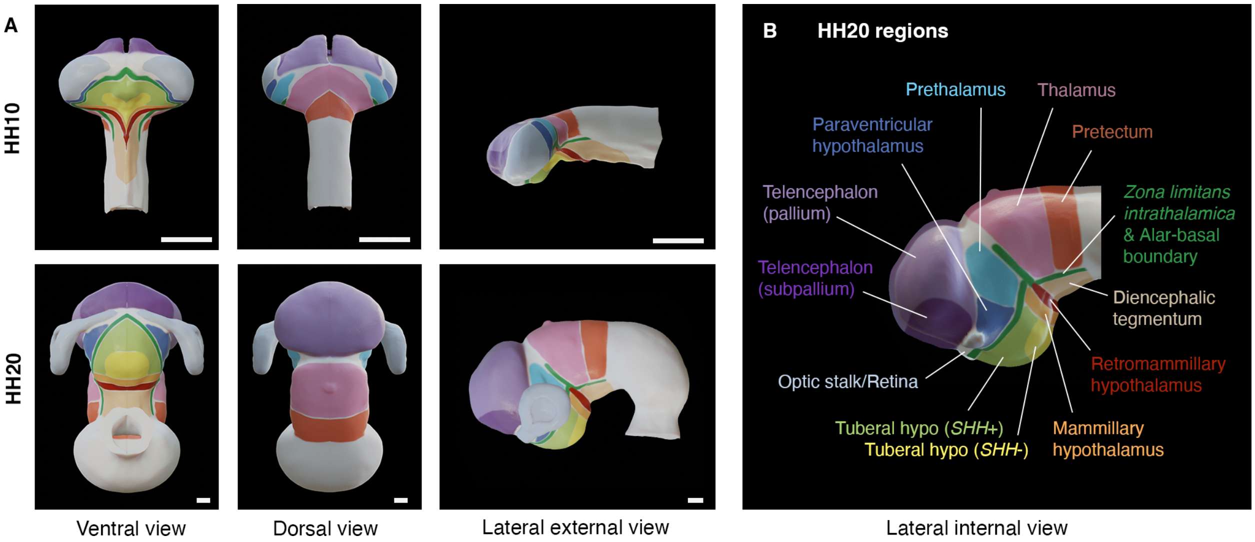

fig. S9

HH10 to HH20 fate map

(A) HH10 fate map and HH20 regions, 4D model. **(B)** HH20 region labels. Scale bars - 250 µm.

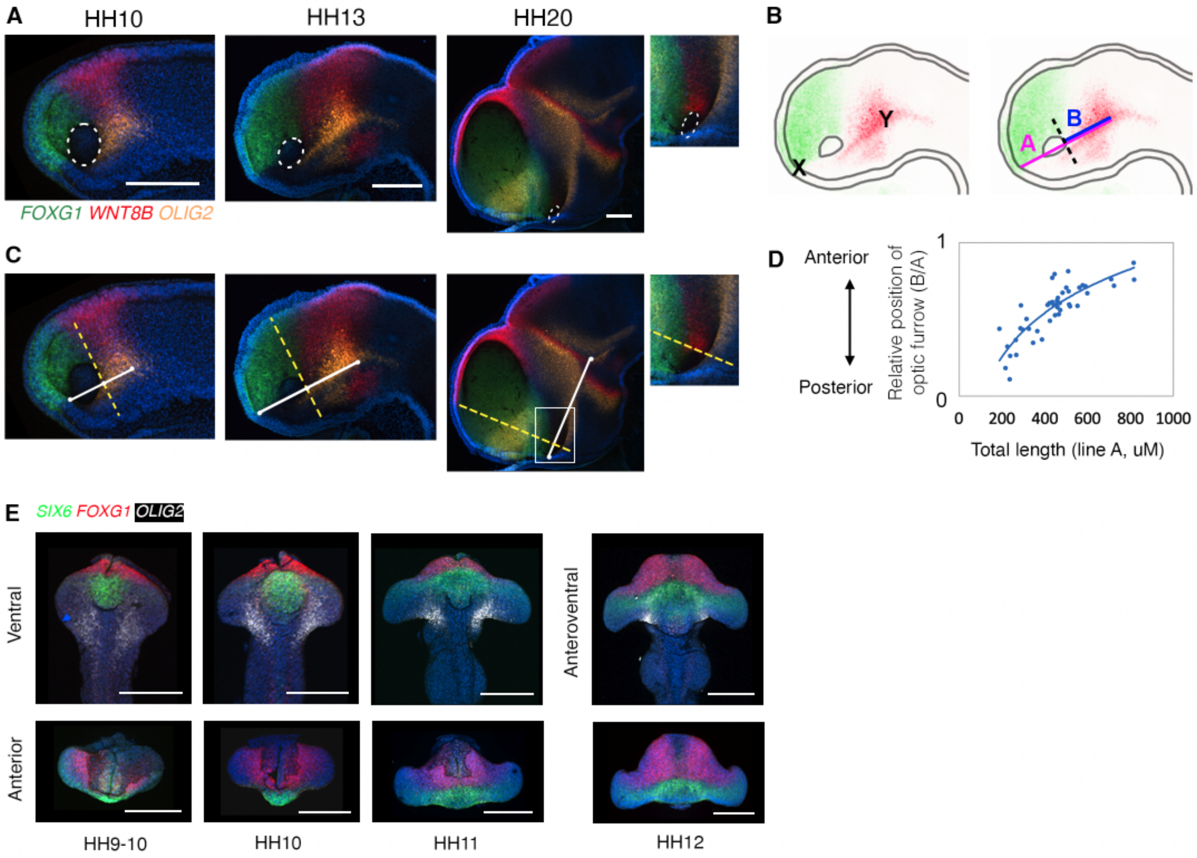

fig. S10

Changing dimensions of the embryonic forebrain

(A-D) The optic stalk draws down to a small anterior-ventral opening. (A, C) Lateral, hemisected view of HH10-20 chick brains following HCR for regional markers. Dotted line in (A) approximates the position of the retinal field at HH10 and optic stalk opening at HH13-HH20. (B-C) Positions of markers/lines used to measure relative A-P position of the posterior optic vesicle/optic stalk, as presented in (D) - see methods for details. **(E)** Dissected neuroepithelia of HH9-12 chicks processed for expression of *SIX6*, *FOXG1* and *OLIG2* by HCR. Scale bars - 250 µm.

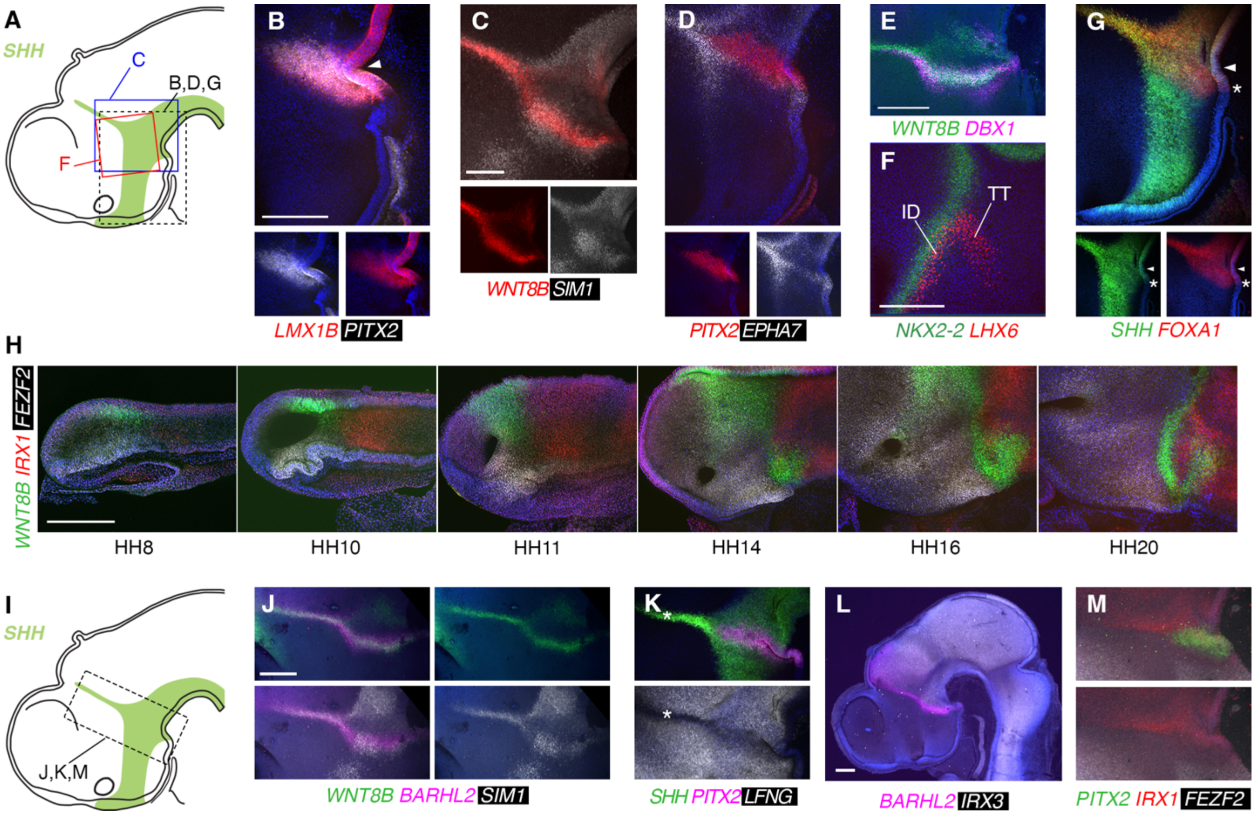

fig. S11

HCR analysis of chick posterior hypothalamus

(A) Regions shown in B-G. **(B-G)** HCR analysis of gene expression patterns in HH20 hemisected chick heads. Arrowhead in (B) and (G) indicates flexure at end of *ARX*$^{+ve}$ floor plate Asterisk in (G) indicates end of *SHH*/*FOXA1*$^{+ve}$ ventral midline. **(H)** Developmental series of HH8-HH20 chicken forebrains analysed by HCR. Hemisected lateral views with the hypothalamus oriented similarly; HH20 example is therefore rotated approximately 90 degrees clockwise compared to (B-G). **(I)** Boxed area indicates region shown in (J-K, M). **(J-M)** HCR analysis of gene expression patterns in HH20 hemisected chick heads. Scale bars - 250 μm.

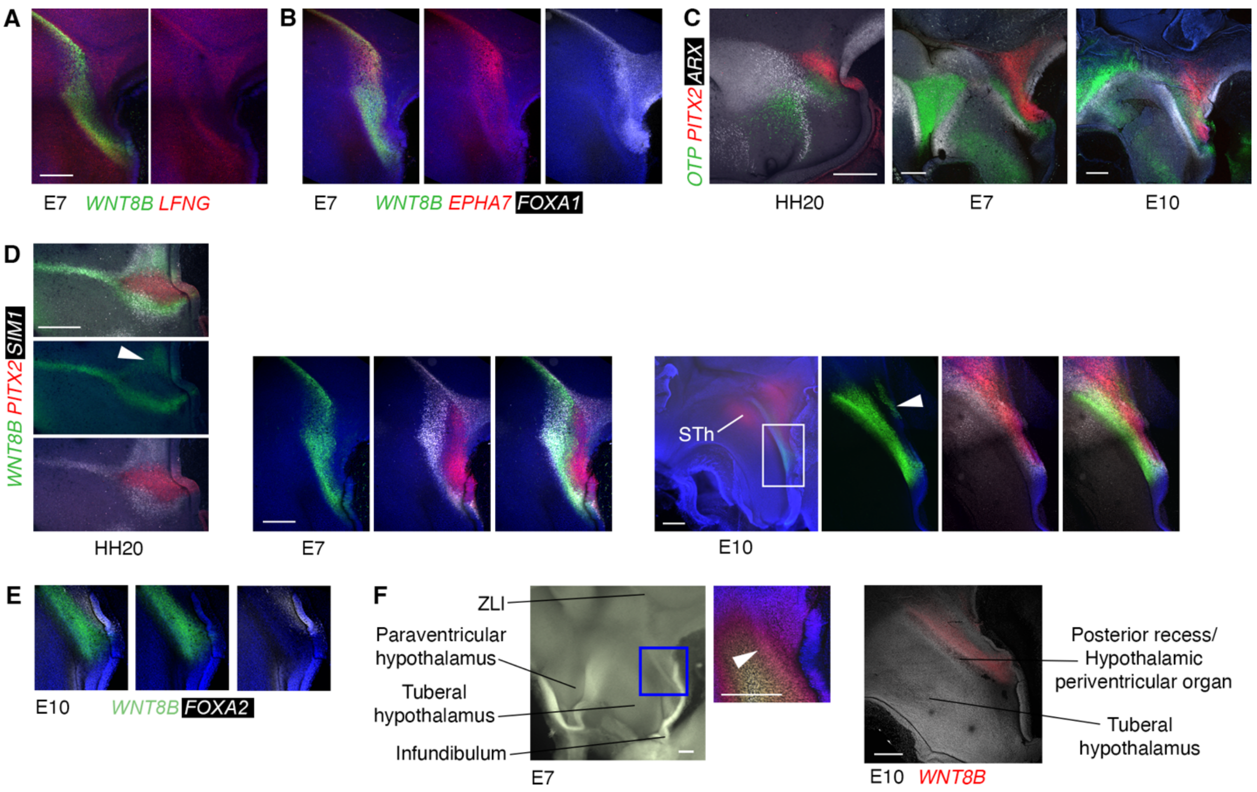

fig. S12

HCR analysis of chick posterior hypothalamus, HH20 - E10

(A, B) E7 chicken ZLI and posterior hypothalamus processed by HCR. **(C, D)** Morphological comparisons of HH20, E7 and E10 chicken hypothalamus, HCR *in situs*. Boxed area in (D) (E10) expanded to right. Arrowheads - weak *WNT8B* expression posterior to *PITX2*$^{+ve}$ domain. **(E)** HCR on E10 sample showing ventral midline *FOXA2* expression. **(F)** Morphological ridge/fold in the E7 and E10 hypothalamus. Boxed area in E7 shown expanded and coloured by z-position to highlight morphology. Same embryo as in (A), arrowhead - ridge. Scale bars - 250 µm.

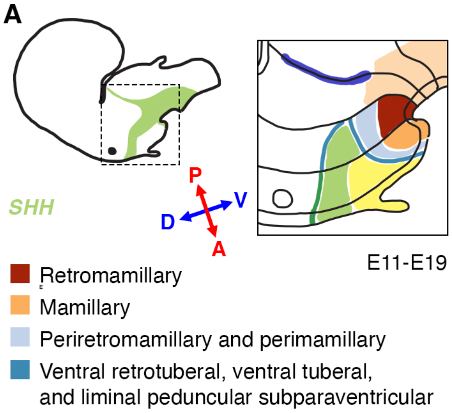

Mouse prosomere domains

	Dorsal		Ventral
Posterior	**Ventral retrotuberal**: Nkx2-1, Lhx6, Arx, Dlx5	**Peri-retromamillary**: Nkx2-1, Otp, Sim1	**Retromamillary**: Pitx2, Foxa1/2, Lmx1a, Irx3, Irx5, Barhl1, Shh; Sim1-negative, Nkx2-1-negative, Foxb1-negative
Anterior	**Ventral tuberal**: Nkx2-1, Lhx6, Arx, Dlx5	**Peri-mamillary**: Nkx2-1, Otp, Sim1	**Mamillary**: Nkx2-1, Sim1, Foxb1, Pitx2 (low); Shh-negative, Foxa1/2-negative

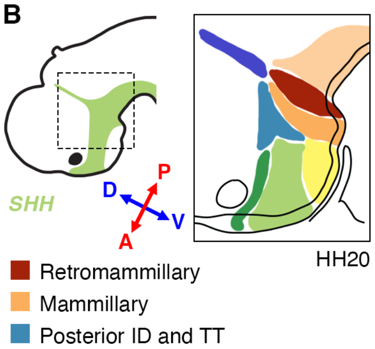

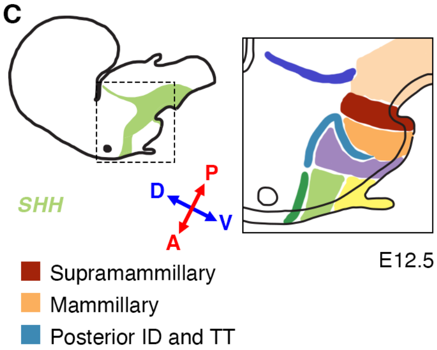

Chicken hypothalamic progenitor domains

Anterior		Posterior
**Tuberomammillary terminal**: NKX2-1, LHX6, ARX, DLX1/2	**Mammillary**: NKX2-1, SIM1, OTP, EMX2, OLIG2	**Retromammillary**: PITX2, FOXA1/2, LMX1B, IRX1, IRX3, SIM1 (low)

Mouse hypothalamic neuronal precursors

Anterior		Posterior
**Tuberomammillary terminal**: Nkx2-1, Lhx6, Arx, Dlx2	**Mammillary**: Nkx2-1, Sim1, Foxb1, Pitx2 (low)	**Supramammillary**: Pitx2, Foxa1, Lmx1a, Irx3, Irx5, Barhl1

fig. S13

Descriptions of hypothalamic progenitor regions in chicken and mouse

(A-C) Hypothalamic progenitor domains, axes, and regional marker genes as described in different species and by different authors. **(A)** Prosomere domains as described for E11-E19 mouse (*2, 3, 4, 5*). Major progenitor regions are colour-coded for ease of comparison, and selected distinguishing marker genes are provided in the tables. Note that the prosomere mamillary, perimamillary and periretromamillary domains together approximately correspond to the mammillary domains in (B) and (C). Outline adapted from (*6*). **(B)** HH20 chicken hypothalamic progenitor domains as described in this study and (*7*), in which the retromammillary domain was referred to as supramammillary. **(C)** E12.5 mouse hypothalamic neuronal precursors, as described in (*8, 9*). ID - Intrahypothalamic Diagonal, TT - Tuberomammillary Terminal, ZLI - *zona limitans intrathalamica*.

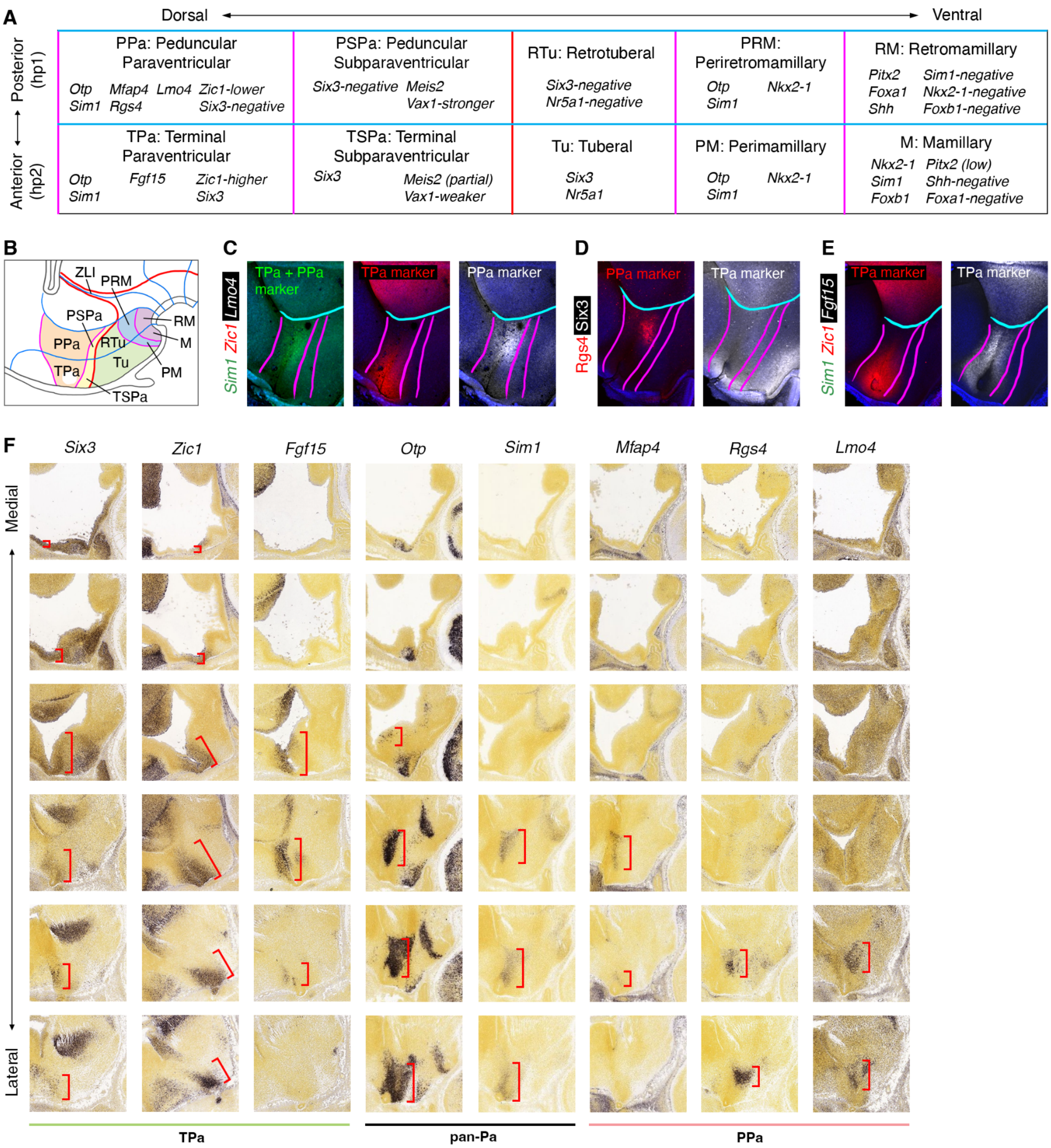

fig. S14

Terminal and peduncular markers show mediolateral differences in the E13 mouse paraventricular hypothalamus

A) Prosomere model hypothalamic regions and identifying marker genes (see Fig. 5D for floor plate detail)(*5*). **B)** Prosomere model, hypothalamic regions highlighted (adapted from (*6*)). **C-E)** HCR-stained E13 mouse forebrains with p3-hp1 (transverse) and selected longitudinal boundaries overlaid. **F)** Allen brain atlas (https://developingmouse.brain-map.org/; experiment ids: *Six3*-100032004; *Zic1*- 100076418; *Fgf15*- 100039971; *Otp*- 100075818; *Sim1*- 100029661;

Mfab4- 100046340; *Rgs4*- 100042374; *Lmo4*- 100054355)(*10*) sagittal sections for Pa marker genes. For each gene, the section nearest the midline, plus the next five serial sections, are shown. Red brackets indicate approximate extent of expression in the Pa zone. Labels at the bottom indicate which prosomere division of the Pa each gene marks. pan-Pa = TPa + PPa.

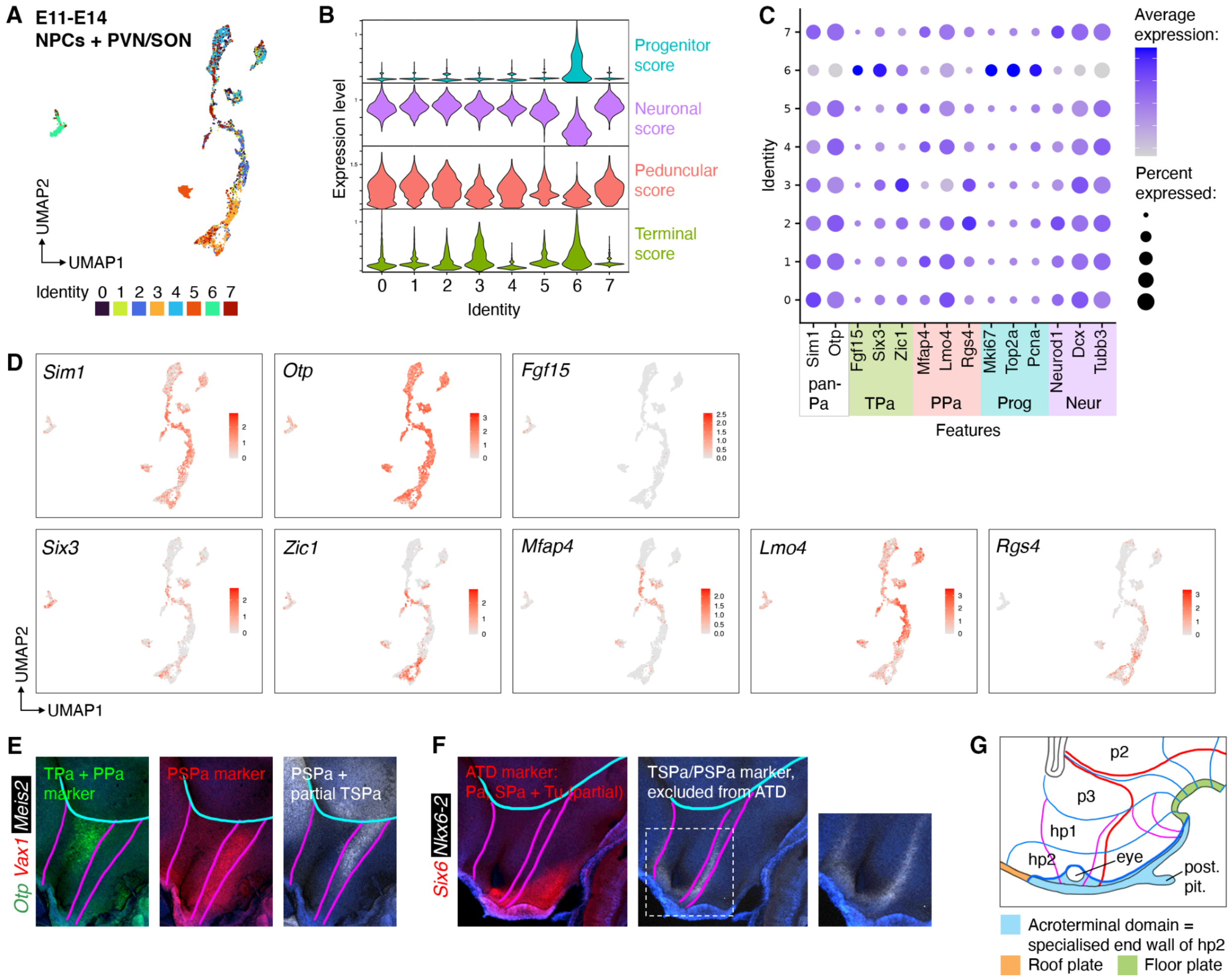

fig. S15

A) UMAP plot, reclustered scRNA-Seq data for E11-14 mouse neural progenitor cells (NPCs) and paraventricular/supraoptic nucleus neurons (PVN/SON) extracted from the main dataset (*11*). **B)** Progenitor, Neuronal, Peduncular and Terminal scores calculated for the eight clusters in (A), using the markers displayed in (C). **C)** Marker gene expression across the eight clusters in (A). **D)** UMAP plots for the dataset in (A) showing Pa/TPa/PPa marker gene expression. **E-F)** HCR-stained E13 mouse forebrains. *Six6* marks the acroterminal domain in the Pa, SPa and dorsal part of the Tu prosomere regions (*5*). **G)** Prosomere model including the acroterminal domain, which gives rise to specialised structures such as the posterior pituitary and optic chiasm. PPa - Peduncular Paraventricular hypothalamus, TPa - Terminal Paraventricular hypothalamus, PSPa - Peduncular Subparaventricular, TSPa - Terminal Subparaventricular, ATD - Acroterminal Domain.

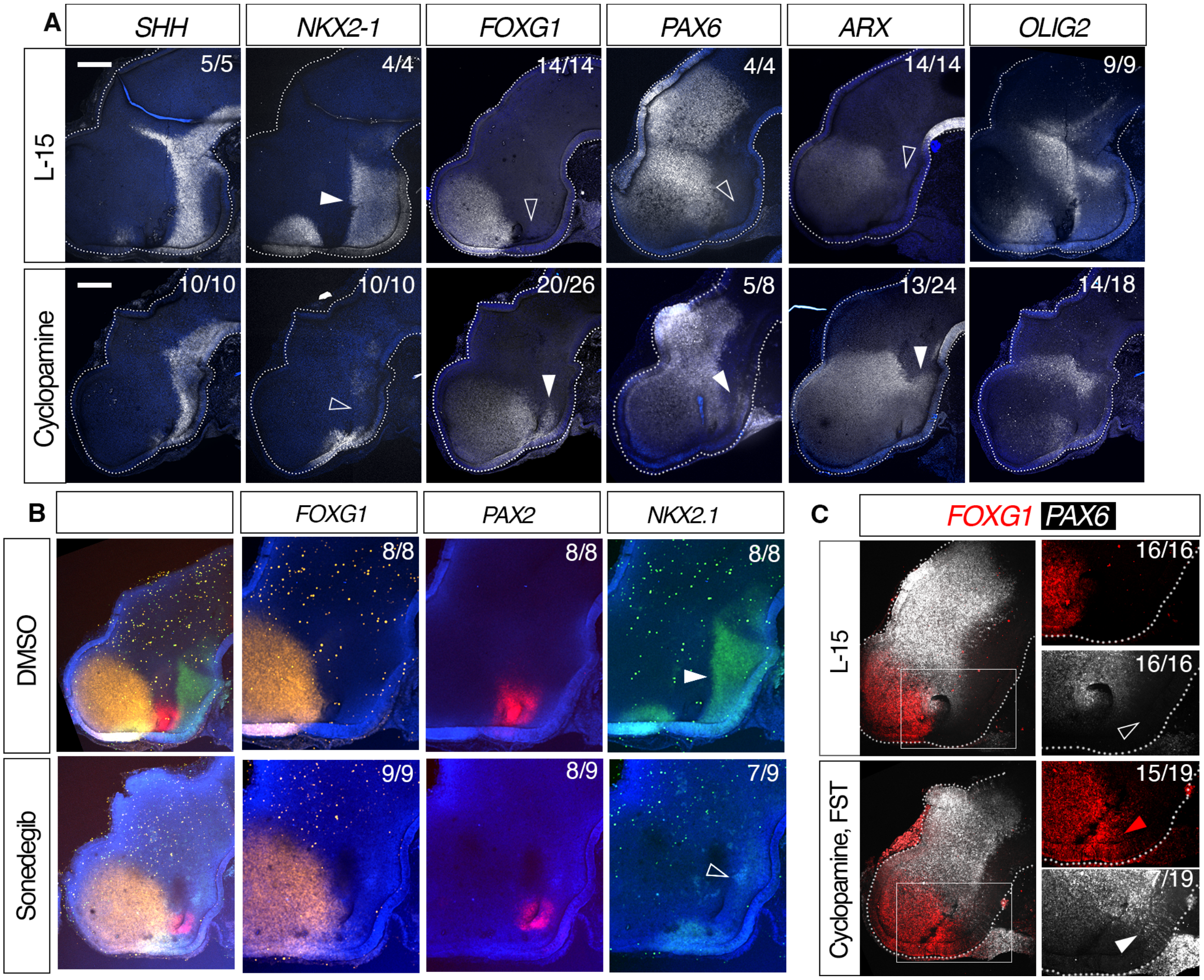

fig. S16

Dorsalisation of the hypothalamus

HH17-18 hemisected chick heads (internal view) after treatment with SHH inhibitor or control medium applied at HH10 and developed for 24 hours. Hemisected heads shown after HCR in situ for markers indicated. (**A**) L-15 control medium (top) or cyclopamine alone (bottom). (**B**) DMSO control (top) or Sonidegib (bottom). (**C**) L-15 control medium (top) or cyclopamine with FST (bottom). After SHH inhibition, the tuberal hypothalamus is reduced in size, *SHH* expression is reduced and *NKX2.1* hypothalamic expression is almost absent (A, C). *FOXG1* remains in the telencephalon and is also expanded into the anterior hypothalamus (A-C). *PAX6* is also ectopically expressed in the ventral tuberal hypothalamus (B, C). *OLIG2* or *ARX* persist in the posterior hypothalamus (C). Closed arrowheads point to regions of expression; open arrowheads to regions where expression cannot be detected. Scale bars - 250 µm. n numbers indicated in top right of each panel (number as represented / number treated per condition). Totals from Cyclopamine-treated, *OLIG2*; and Cyclopamine, FST co-treated samples are pooled from 2 independent repeats; Cyclopamine-treated, *ARX* and *FOXG1* are pooled from 3 repeats.

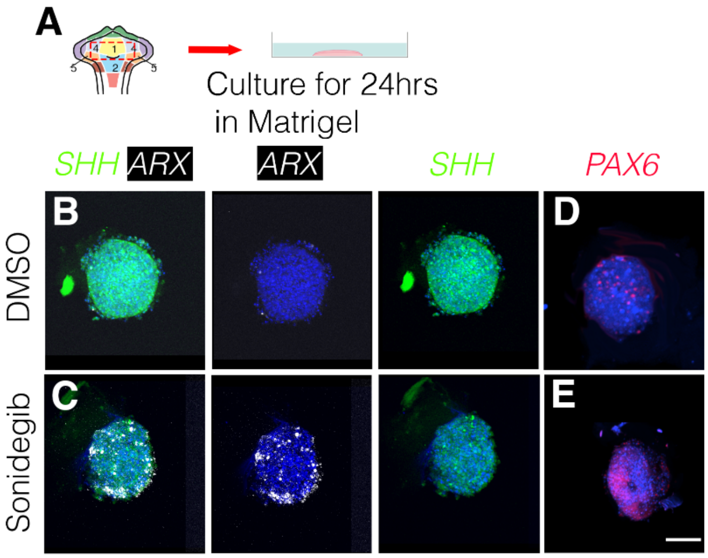

fig. S17:

***Ex vivo* explant analysis of dorsalisation**

(A) Schematic showing the region explanted at HH10 and cultured with DMSO or Sonidegib for 24hrs. (B-E) Maximum intensity projection of HH10 explant treated with DMSO or Sonidegib and cultured for 24hrs. (B-E) Control DMSO and Sonidegib treated explants labelled for *SHH/ARX* (B,C) and *PAX6* (D,E). After SHH inhibition, explants show increased expression of *ARX* (C) and *PAX6* (E). n=3-5 explants per condition. Scale bars - 250 µm

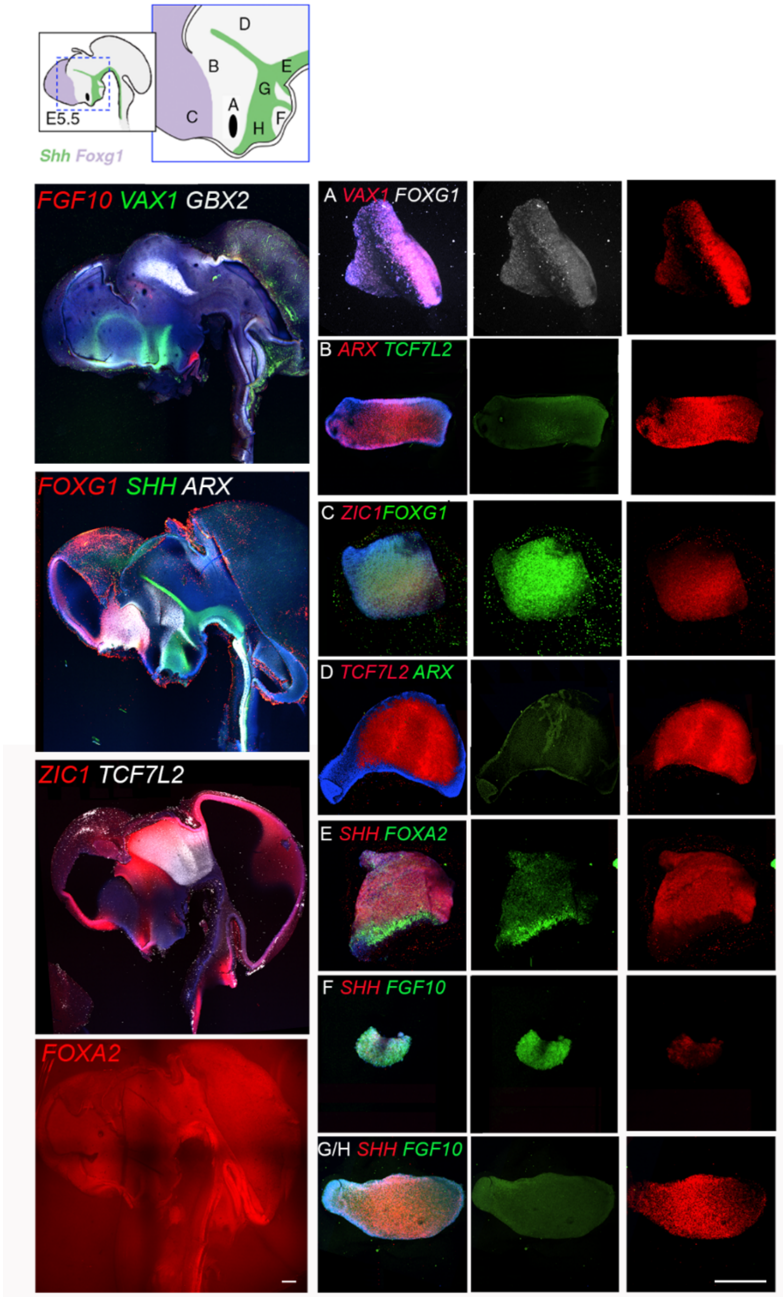

fig. S18

Accurate dissection of forebrain regions

Top schematics indicate regions dissected: A, PV hypothalamus; B, prethalamus; C, telencephalon; D, thalamus; E, diencephalic midline/basal plate; F, *SHH*$^{-ive}$ tuberal hypothalamus; G, H, posterior (G) and anterior (H) regions of *SHH*$^{+ive}$ basal hypothalamus. Left hand panel: E5.5 hemiviews (internal view) after HCR labelling with selected markers. Right hand panels: double in situ or single channel views of isolated forebrain regions A-G, at t = 0hr confirms accuracy of dissection. Scale bars: 250 µm

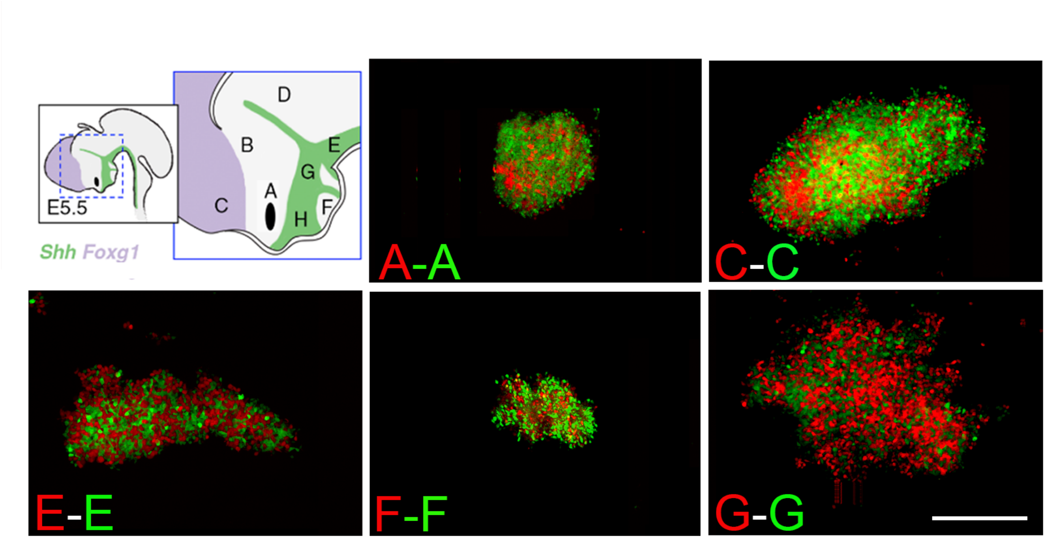

fig. S19

Cells from the same region intersperse

In vitro cultures 48 hours after dissection, disaggregation and mixing of transgenic GFP- and RFP-expressing cells from regions in schematics. Scale bars: 100 µm

Supplementary references:

1. Oh, J. D. H., et al. Insights into digit evolution from a fate map study of the forearm using Chameleon, a new transgenic chicken line. *Development* **151** (2024).

2. Puelles L., Martinez-De-La-Torre M., Bardet S., Rubenstein J.L.R. 'Hypothalamus', pp. 221-309, in: *The Mouse Nervous System*, C. Watson *et al*. Editor, (*Elsevier Science & Technology 2011*).

3. Puelles L., Rubenstein J. L. R. A new scenario of hypothalamic organization: rationale of new hypotheses introduced in the updated prosomeric model. *Front Neuroanat* **9**, 27 (2015).

4. Diaz C., Puelles L. Developmental Genes and Malformations in the Hypothalamus. *Front. Neuroanat.* **14**, 607111 (2020).

5. Ferran J., Puelles L., Rubenstein J. L. R. Molecular codes defining rostrocaudal domains in the embryonic mouse hypothalamus. *Front. Neuroanat.* **9**, 46 (2015).

6. Puelles L., Martinez-De-La-Torre M., Bardet S., Rubenstein J.L.R. 'Hypothalamus', in: *The Mouse Nervous System*, C. Watson *et al*. Editor, (Elsevier Science & Technology 2012), pp. 221-309.

7. Kim D. W., et al. Single-cell analysis of early chick hypothalamic development reveals that hypothalamic cells are induced from prethalamic-like progenitors. *Cell Rep.* **38**, 110251 (2022).

8. Shimogori T., et al. A genomic atlas of mouse hypothalamic development. *Nat. Neurosci.* **13**, 767–775 (2010).

9. Kim D. W., et al. The cellular and molecular landscape of hypothalamic patterning and differentiation from embryonic to late postnatal development. *Nat. Commun.* **11**, 4360 (2020).

10. Allen Institute for Brain Science (2004). Allen Mouse Brain Atlas [dataset]. Available from https://developingmouse.brain-map.org *Allen Institute for Brain Science* (2011).

11. Kim D. W., et al. Decoding gene networks controlling hypothalamic and prethalamic neuron development. *Cell Rep*. **44**(6):115858 (2025).
