# Supplementary material for: Resolving forebrain developmental organisation by analysis of differential growth patterns
Source: Nat Commun. 2025 Dec 21;17:901. doi: 10.1038/s41467-025-67623-6 (PMC12830808; doi:10.1038/s41467-025-67623-6)
Supplement: Supplementary file 2 — Description of Additional Supplementary Files [file 41467_2025_67623_MOESM2_ESM.pdf]

Description of Additional Supplementary Files

File name: Supplementary Movie 1

Description: **Fate Map, chicken neural tube, Hamburger-Hamilton (HH) stages 10 to 20.** Fate map compiled from DiI and grafting studies, analysed at HH20, HH23-25 and HH35-40. Transitions between stages are visualised based on neuroectoderm morphology at HH10, HH11, HH14 and HH20.

File name: Supplementary Movie 2

Description: **'Digital dye' spots on our model recapitulate key growth patterns seen *in vivo*.** Digital dye spots on HH10 neurectoderm visualised at different angles and 'developed' to HH20. Resulting growth lines are compared to examples of in vivo injections at HH10 following development to HH18-20.

File name: Supplementary Movie 3

Description: **Growth patterns shaping the anterior hypothalamus and the dorsal forebrain anterior to the ZLI.** Description of how growth patterns in the anterior hypothalamus and dorsal forebrain contribute to forebrain and eye morphogenesis.

File name: Supplementary Movie 4

Description: **Growth patterns shaping the posterior hypothalamus and the dorsal forebrain posterior to the ZLI.** Observations on growth lines and morphogenesis in forebrain regions posterior to the *zona limitans intrathalamica*.
