# Supplementary material for: Resolving forebrain developmental organisation by analysis of differential growth patterns
Source: Nat Commun. 2025 Dec 21;17:901. doi: 10.1038/s41467-025-67623-6 (PMC12830808; doi:10.1038/s41467-025-67623-6)
Supplement: Supplementary file 7 — Reporting Summary [file 41467_2025_67623_MOESM7_ESM.pdf]

nature portfolio

Corresponding author(s): Marysia Placzek
Last updated by author(s): 11/18/2025

Reporting Summary

Nature Portfolio wishes to improve the reproducibility of the work that we publish. This form provides structure for consistency and transparency in reporting. For further information on Nature Portfolio policies, see our Editorial Policies and the Editorial Policy Checklist.

Statistics

For all statistical analyses, confirm that the following items are present in the figure legend, table legend, main text, or Methods section.

n/a	Confirmed	
☐	☒	The exact sample size (*n*) for each experimental group/condition, given as a discrete number and unit of measurement
☐	☒	A statement on whether measurements were taken from distinct samples or whether the same sample was measured repeatedly
☐	☒	The statistical test(s) used AND whether they are one- or two-sided<br>*Only common tests should be described solely by name; describe more complex techniques in the Methods section.*
☒	☐	A description of all covariates tested
☒	☐	A description of any assumptions or corrections, such as tests of normality and adjustment for multiple comparisons
☐	☒	A full description of the statistical parameters including central tendency (e.g. means) or other basic estimates (e.g. regression coefficient) AND variation (e.g. standard deviation) or associated estimates of uncertainty (e.g. confidence intervals)
☐	☒	For null hypothesis testing, the test statistic (e.g. *F*, *t*, *r*) with confidence intervals, effect sizes, degrees of freedom and *P* value noted<br>*Give P values as exact values whenever suitable.*
☒	☐	For Bayesian analysis, information on the choice of priors and Markov chain Monte Carlo settings
☒	☐	For hierarchical and complex designs, identification of the appropriate level for tests and full reporting of outcomes
☒	☐	Estimates of effect sizes (e.g. Cohen's *d*, Pearson's *r*), indicating how they were calculated

Our web collection on statistics for biologists contains articles on many of the points above.

Software and code

Policy information about availability of computer code

Data collection	Data was collected by microscopy. Zeiss Axiovision software (ZEN2Pro (blue edition); Leica LASX software (LASX1.1.0.12420); Zeiss ZEN software (blue edition); Nikon Elements (NIS-Elements AR version 5.42.03) software.
Data analysis	Image analysis was performed in FIJI (ImageJ) version 2.7.0/1.53t and Adobe Photoshop 2022. Modelling of forebrain development was performed using Blender versions 3 and 4 (blender.org). Statistical tests were performed using GraphPad Prism 10.6.1

For manuscripts utilizing custom algorithms or software that are central to the research but not yet described in published literature, software must be made available to editors and reviewers. We strongly encourage code deposition in a community repository (e.g. GitHub). See the Nature Portfolio guidelines for submitting code & software for further information.

Data

Policy information about availability of data

All manuscripts must include a data availability statement. This statement should provide the following information, where applicable:

- Accession codes, unique identifiers, or web links for publicly available datasets
- A description of any restrictions on data availability
- For clinical datasets or third party data, please ensure that the statement adheres to our policy

The authors declare that the minimum dataset that is necessary to interpret, verify and extend the research in this article is included within the manuscript and its supplementary information files. All scRNA-Seq data used in this study were previously generated and published in Kim et al. (2025), and deposited in the NCBI Gene

Expression Omnibus (GEO) under accession number GSE284492. Source data are provided with this paper.

Research involving human participants, their data, or biological material

Policy information about studies with human participants or human data. See also policy information about sex, gender (identity/presentation), and sexual orientation and race, ethnicity and racism.

Reporting on sex and gender	No sex and gender-based analyses were performed, as the great majority of studies were performed on embryos prior to stages of sexual differentiation.
Reporting on race, ethnicity, or other socially relevant groupings	N/A
Population characteristics	N/A
Recruitment	N/A
Ethics oversight	N/A

Note that full information on the approval of the study protocol must also be provided in the manuscript.

Field-specific reporting

Please select the one below that is the best fit for your research. If you are not sure, read the appropriate sections before making your selection.

[x] Life sciences [ ] Behavioural & social sciences [ ] Ecological, evolutionary & environmental sciences

For a reference copy of the document with all sections, see nature.com/documents/nr-reporting-summary-flat.pdf

Life sciences study design

All studies must disclose on these points even when the disclosure is negative.

Sample size	Sample sizes are based on previous studies, in the neurodevelopment field, that provide descriptive and comparative data in similar experiments. Over decades of work, reproduced by others, numbers of individual samples/animals examined to achieve statistical significance is normally ~5-6 embryos or explants per condition. Significance is based on consistency of observations, both within an animal and between stage-matched animals, or explants. All data is based on a 'strong' phenotype. Quantitation is performed wherever possible.
Data exclusions	Embryos that develop abnormally - ie have gross abnormalities independent of clear phenotypes (eg cyclopia) are excluded
Replication	Experiments were replicated as follow: fate mapping studies (Figure 1) each zone and associated boundaries targeted on at least 5 different days; mixing experiments (Figure 6E) replicated three times; dorsalisation experiments replicated as follows: cyclopamine replicated six times; Fst and cyclopamine replciated once, Sonedigib replicated twice. All attempts at replication were successful. Note that generally, experiments are performed by 2 individuals.
Randomization	Embryos were randomly assigned for in vivo and ex vitro experiments.
Blinding	All analyses are verified by a blinded independent observer.

Reporting for specific materials, systems and methods

We require information from authors about some types of materials, experimental systems and methods used in many studies. Here, indicate whether each material, system or method listed is relevant to your study. If you are not sure if a list item applies to your research, read the appropriate section before selecting a response.

Materials & experimental systems

n/a	Involved in the study	
[ ]	[x]	Antibodies
[x]	[ ]	Eukaryotic cell lines
[x]	[ ]	Palaeontology and archaeology
[ ]	[x]	Animals and other organisms
[x]	[ ]	Clinical data
[x]	[ ]	Dual use research of concern
[x]	[ ]	Plants

Methods

n/a	Involved in the study	
[x]	[ ]	ChIP-seq
[x]	[ ]	Flow cytometry
[x]	[ ]	MRI-based neuroimaging

Antibodies

Antibodies used	Rabbit anti-laminin α (LAMA), Sigma Product number L9393 batich number 092M4759 at 1:1000
Validation	Yukiko Nakaya, Erike W Sukowati, Cantas Alev, Fumie Nakazawa, Guojun Sheng Involvement of dystroglycan in epithelial-mesenchymal transition during chick gastrulation. Cells, tissues, organs (2010-11-06)

Animals and other research organisms

Policy information about studies involving animals; ARRIVE guidelines recommended for reporting animal research, and Sex and Gender in Research

Laboratory animals	Fertilised Bovan Brown eggs (Henry Stewart & Co., Norfolk, UK), and transgenic Chameleon (cytbow) (1), Cytoplasmic GFP (2), and Flamingo (tdTomato) (3) eggs (The Roslin Institute, Edinburgh, UK) were incubated in a humidified incubator at 37°C until the desired stage, according to (4,5). Time-mated pregnant wildtype C57B1/6 mice were sourced from Envigo RMS (UK) Limited, Shaws Farm, Bicester.
Wild animals	N/A
Reporting on sex	No sex and gended-based analyses were performed, as the majority of studies were performed on embryos prior to sexual differentiation.
Field-collected samples	N/A
Ethics oversight	All studies and procedures were conducted at our licensed establishment (UK Hom Office under the UK Animals (Scientific Procedures) Act 1986) and were approved by the University of Sheffield Animal Welfare and Local Ethical Review committee (AWERB). Named Animal Care and Welfare Officers (NACWOs) had oversight of all incubated eggs.

Note that full information on the approval of the study protocol must also be provided in the manuscript.

Plants

Seed stocks	N/A
Novel plant genotypes	N/A
Authentication	N/A
